# Accelerating High‐Fidelity Airfoil Design via Physics‐Informed Video Diffusion Model

**DOI:** 10.1002/advs.78532

**Published:** 2026-11-03

**Authors:** Yifeng Ai, Yijia Guo, Hongfu Zhang, Lei Zhou, Tiejun Huang, Weiqiu Chen, Xuhui He, Peng Guo, C. W. Lim, Lei Ma

**Affiliations:** ^1^ City University of Hong Kong Hong Kong China; ^2^ National Key Laboratory for Multimedia Information Processing Peking University Beijing China; ^3^ The Hong Kong Polytechnic University Hong Kong China; ^4^ Central South University Changsha China; ^5^ Zhejiang University Hangzhou China; ^6^ National Biomedical Imaging Center Peking University Beijing China

**Keywords:** accelerated inverse design, computational fluid dynamics, diffusion model, direct numerical simulation, generative artificial intelligence

## Abstract

Airfoil design remains a challenging multi‐objective optimization problem requiring precise geometric control for optimal aerodynamic performance. Data‐driven inverse design methods mitigate this complexity but often yield non‐physical results when physical constraints are not explicitly incorporated. Here, we construct a high‐fidelity NACA–Nek1000 datasets with 53 400 samples from direct numerical simulations to train a dual physics‐informed video diffusion model (PVDM). The governing equations of incompressible flow are intrinsically coupled with the diffusion learning process, enabling the reconstruction of full‐field velocity and pressure distributions from lift‐to‐drag response sequences. The PVDM achieves accurate low‐Reynolds number airfoil reconstruction and real‐time flow generation within 30 s per case, approximately 600 times faster than conventional simulations, while maintaining over 90% fidelity. The reconstructed airfoils are further modeled as propeller geometries and experimentally validated, which well fit the simulated lift‐to‐drag response, establishing a rapid and generalizable framework for high‐fidelity, physics‐informed inverse aerodynamic design.

## Graphical abstract

Physics‐informed video diffusion enables rapid inverse aerodynamic design by mapping target lift‐to‐drag response to airfoil geometries and full‐field flow distributions. Trained on 53 400 DNS samples, the model achieves over 90% fidelity with ∼30 s inference, while experimentally realized propeller designs demonstrate a practical pathway from aerodynamic targets to manufacturable structures.

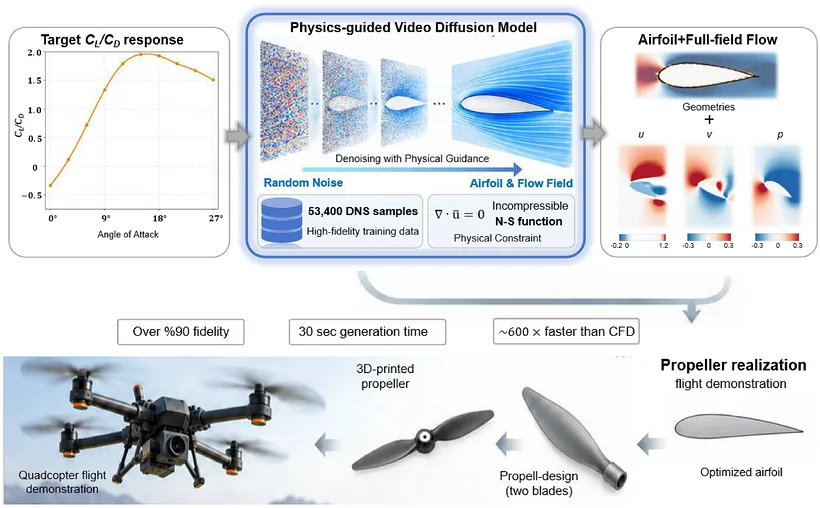

## 1 Introduction

Airfoil design constitutes a fundamental challenge in micro‐aircraft and underwater engineering, representing a complex, multi‐objective optimization task. This process centers on optimizing the lift‐to‐drag ratio (*C_L_*/*C_D_*) to ensure optimal aerodynamic efficiency. However, achieving optimal *C_L_*/*C_D_* performance across a wide range of angles of attack (AoAs) requires intricate geometric balancing. Precise tuning of parameters, specifically thickness distribution, camber magnitude, and maximum camber position are critical, as each exerts a pronounced non‐linear influence on the flow field. Historically, aerodynamic characteristics are typically evaluated through computationally intensive fluid dynamics simulations, ranging from high‐fidelity direct numerical simulation (DNS) [1, 2, 3], large‐eddy simulation (LES) [4, 5, 6] to efficient Reynolds‐averaged Navier–Stokes (RANS) models [7, 8, 9], or through experimental wind‐tunnel validation using particle image velocimetry [10, 11, 12, 13]. Despite these strict requirements and high computational costs, the current, largely heuristic inverse design workflow, including gradient‐based [14] and gradient‐free optimization algorithms [15, 16], remains highly time‐consuming, computationally demanding, and reliant on specialized knowledge, ultimately yielding only incremental performance improvements [17].

To overcome these limitations, Deep Learning is now harnessed to learn implicit design constraints and latent representations, fundamentally augmenting computational design capabilities. Methods like Convolutional Neural Networks [18] and Reinforcement Learning [19] expedite the iterative optimization of aerodynamic profiles, substantially mitigating the computational burden of traditional high‐fidelity simulation. However, these optimization approaches remain reliant on multi‐step and sequential refinement. Inverse design strategies have emerged to mitigate this computational latency. These approaches directly infer geometric profiles from target performance metrics, such as lift (*C_L_*) and drag (*C_D_*) coefficients (normalized by chord length), spanning diverse flow regimes [20]. Crucially, these inverse approaches often rely on purely correlational, sequence‐to‐sequence mappings that bypass the underlying physical laws, thereby fundamentally compromising physical interpretability and introducing significant design ambiguity for domain experts. Architectural constraints further restrict them to geometry synthesis without flow field co‐prediction, a critical omission that impedes subsequent optimization and rigorous validation protocols.

This study introduces a physics‐informed video diffusion model [21, 22, 23, 24] (PVDM) for accurate reconstructing of low‐Reynolds number airfoil geometries and generating corresponding flow field results through a mechanical process such as the incremental AoAs of the airfoil. Airfoils are parameterized by the three defining NACA variables: maximum camber, camber position, and chord thickness. The model infers these geometric features from *C_L_*/*C_D_* sequences [25] across varying AoA values. Training and testing use the NACA–Nek1000 datasets, comprising 53 400 DNS samples of two‐dimensional incompressible flow fields generated via high‐order spectral element simulations at three spatial resolutions. The *C_L_*/*C_D_* response tokens are extracted from DNS and used as model inputs. Physics guidance is incorporated by embedding normal gradients of the flow field into the diffusion framework, coupling the learning process with the incompressible Navier–Stokes equations. The model adopts a dual PVDM architecture based on a 3D U‐Net with cross‐attention layers that inject *C_L_*/*C_D_*–AoA sequences into spatial and temporal dimensions. One branch reconstructs global flow contours, and the other refines near‐airfoil regions, compensating for the limitations of single 3D U‐Net models in resolving high‐resolution features.

The proposed PVDM generates flow‐field videos within 30 s per case, approximately 600 times faster than DNS (∼300 min for 10 cases) while maintaining > 90% structural fidelity (PSNR: 34–49 *dB*; SSIM: 0.83–0.99). It requires only *C_L_*/*C_D_* sequences as inputs, eliminating boundary condition setup and pre‐testing overhead. The results demonstrate high temporal and spatial coherence, establishing a scalable paradigm for physics‐informed generative modeling in aerodynamic inverse design. By achieving a formidable acceleration relative to high‐fidelity DNS, the approach effectively resolves the chronic computational bottleneck associated with optimizing complex flow fields. This unparalleled efficiency transforms the traditionally intractable problem of *C_L_*/*C_D_*‐guided inverse design into a practical, iterative workflow, facilitates real‐time design loops and seamless integration into digital twin environments. The PVDM model holds the potential to break the incremental design barrier by enabling global optimization searches across complex, high‐dimensional spaces, fostering radical innovation in inverse aerodynamic design.

## 2 Materials and Methods

### 2.1 Training and Testing Datasets Generation

The construction of the NACA‐Nek1000 dataset relies on a rigorous pipeline linking geometric parameterization to high‐fidelity flow physics, as summarized in Figure 1. To ensure comprehensive coverage of the design space, we generated airfoil geometries based on the NACA 4‐digit parameterization, sweeping through a wide range of camber, thickness, and angle of attack configurations (Figure 1a).

**FIGURE 1:**
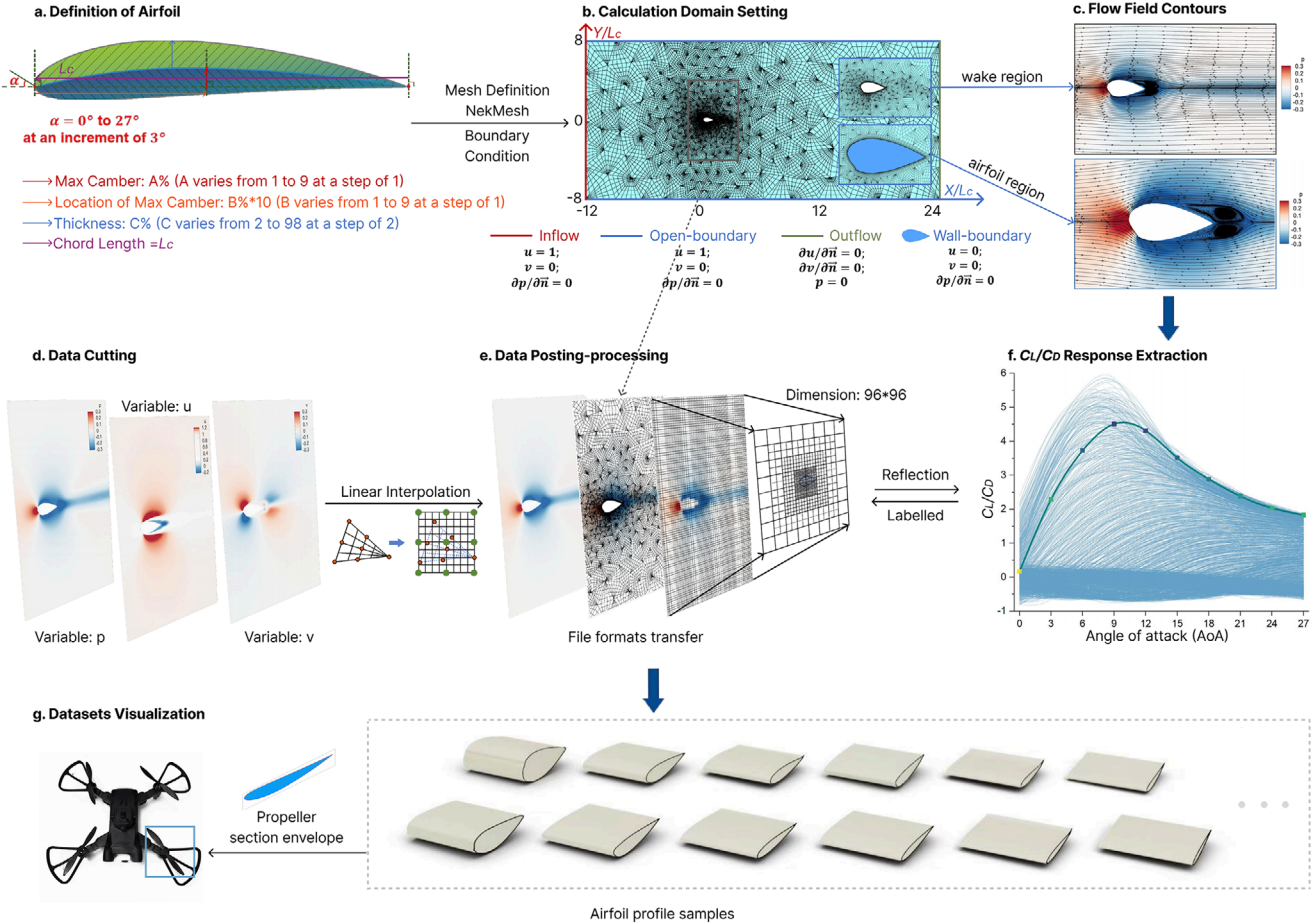
Process employed for generating the training and testing datasets: **(a) Definitions of Airfoil**: airfoil envelopes follow the NACA‐4 digit, maximum camber A varies from 1 to 9 at a step of 1, location of max camber B%*10 varies from 1 to 9 at a step of 1, thickness C% varies from 2 to 98 at a step of 2, chord length *L_c_*; **(b) Calculation Domain Settings**: the mesh is densified significantly around the airfoil and the wake region for promoting the accuracy, the domain area is set as *X/L_c_* varies from ‐12 to 24, *Y/L_c_* varies from ‐8 to 8. The boundary conditions: inflow side as well as two sides is set as *u = U*, *v =* 0 and $\frac{\partial p}{\partial \vec{n}}$ *=* 0 ($\vec{n}$ is the unit normal vector of the boundary), outflow is set as $\frac{\partial u}{\partial \vec{n}}$ *=* 0, $\frac{\partial v}{\partial \vec{n}}$ and *p* *=* 0, the airfoil wall boundary is set as *u =* 0, *v =* 0, and $\frac{\partial p}{\partial \vec{n}}$ *=* 0; (c) Flow Field Contours: The pressure contours are superimposed with velocity streamlines of airfoil region and wake region; (d) Data Cutting: flow fields of *u*, *v* and *p* are cut out which focus on the target region of flow past the airfoil; (e) Data Post‐processing: the cut‐out flow fields are linearly interpolated into 96 × 96 structured data and transferred into files as the “npy” format; (f) *C_L_*/*C_D_* Response Extraction: the mean *C_L_*/*C_D_* response is extracted from the wall surface of airfoil, which labels the corresponding “npy” file of flow field and build accurate reflection; (g) Datasets Visualization: the training protocol comprises 1800 airfoil profiles which can be visualized by the span‐wise fixed airfoil. Note that all the variables in this paper are dimensionless, which are normalized by the reference velocity *U* and chord length *L_c_*.

The high‐fidelity flow field of such airfoil profiles is calculated by DNS at Reynolds number *Re*  =  1000 (*Re*  =  *UL_c_*/*v*, where the inlet flow velocity *U*  =  1*m*/*s*, chord length *L_c_* =  0.1*m*, *v* is the kinematic viscosity). We employed a higher‐order spectral‐element method with a self‐adaptive meshing strategy to guarantee results accuracy, particularly in the critical near‐wall and wake regions (Figure 1b,c). To standardize the data for learning, the resolved two‐velocity‐components (*u*, *v*) and pressure (*p*) were cropped and interpolated onto uniform 96 × 96 grids, stored in floating‐point format to preserve numerical precision (Figure 1d,e). Note that all the variables in this paper are dimensionless, which are normalized by the reference velocity *U* and chord length *L_c_*. By pairing each full‐field flow snapshot with its corresponding lift and drag coefficients, *C_L_* and *C_D_* (Figure 1f), we constructed a high‐fidelity training dataset comprising 53 400 paired samples (Figure 1g). The dataset spans 1780 airfoil geometries, with ten angles of attack (AoAs; 0°–27° in 3° increments) treated as a complete sequence for each geometry. To preserve the integrity of the aerodynamic parameter space, only complete AoA sequences were included, and no airfoil geometry was represented by a partially sampled subset of incidence angles. To further enforce consistency with the governing Navier–Stokes equations, the two velocity components, *u* and *v*, together with the pressure field *p*, were jointly incorporated into the physics‐informed constraints of the DDPM. Further mesh and *C_L_/C_D_* curve validation details are displayed in Section S2.3 and S2.4 (Table S4 and Figures S2 and S3).

### 2.2 Physics‐Informed Video Diffusion Model

Figure 2a shows the training phase of the proposed PVDM. We first perform the tokenization process, in which the *C_L_*/*C_D_* values varying with the AoA are encoded as a temporal vector to label each frame. Before temporal embeddings are applied, the shaped blank frames are spatially convolved and appended with spatial attention. These spatio‐temporal features are then passed into the 3D U‐Net architecture to predict noise at time *t* for the DDPM process. The forward process of DDPM can be mathematically represented for a process transitioning over a period *T*. For this model, the Gaussian noise is gradually added to the data, which is later denoised in a learned reverse process seeking to reconstruct the original distribution. The probability of the distribution *q* from point *x_t_*_−1_ at time *t−*1 to *x_t_* at time step *t*, which remains to be learned, is a fixed Markov chain with a given variance schedule:

(1) $$q\left(x_{t}|x_{t-1}\right)=N\left(x_{t};\sqrt{1-\beta_{t}}x_{t},\beta_{t}I\right),t\in \left\{0,1,\text{…},T-1\right\}$$

where *x_1_, …, x_T_* are the latents of the same dimensionality as the data *x_0_* ∼ *q*(*x_0_*), β_*t*_∈ (0,1) is the scheduled variance associated with the Gaussian distribution N, and *I* denotes the identity matrix. The closed‐form sampling process of Equation 1 is:

(2) $$x_{t}=\sqrt{{\bar{\alpha }}_{t}}\;x_{0}+\sqrt{1-{\bar{\alpha }}_{t}}z$$

where z ∼ (0,1) is a noise‐predictor and ${\bar{a}}_{t}=\prod_{i=0}^{t}\left(1-\beta_{t}\right)$. To train this model, we can optimize the variational lower‐bound for *p*_θ_(*x*_0_), which approximates the reverse transitions of the forward process *q*. In actual training, we simply set the optimization objective as:

(3) $$L_{simple}=E_{t∼\left[1,T\right],x_{0}∼q\left(x_{0}\right),z∼N\left(0,1\right)}\;\left[∥z-z_{\theta }\left(x_{0},t\right)∥\right]$$

**FIGURE 2:**
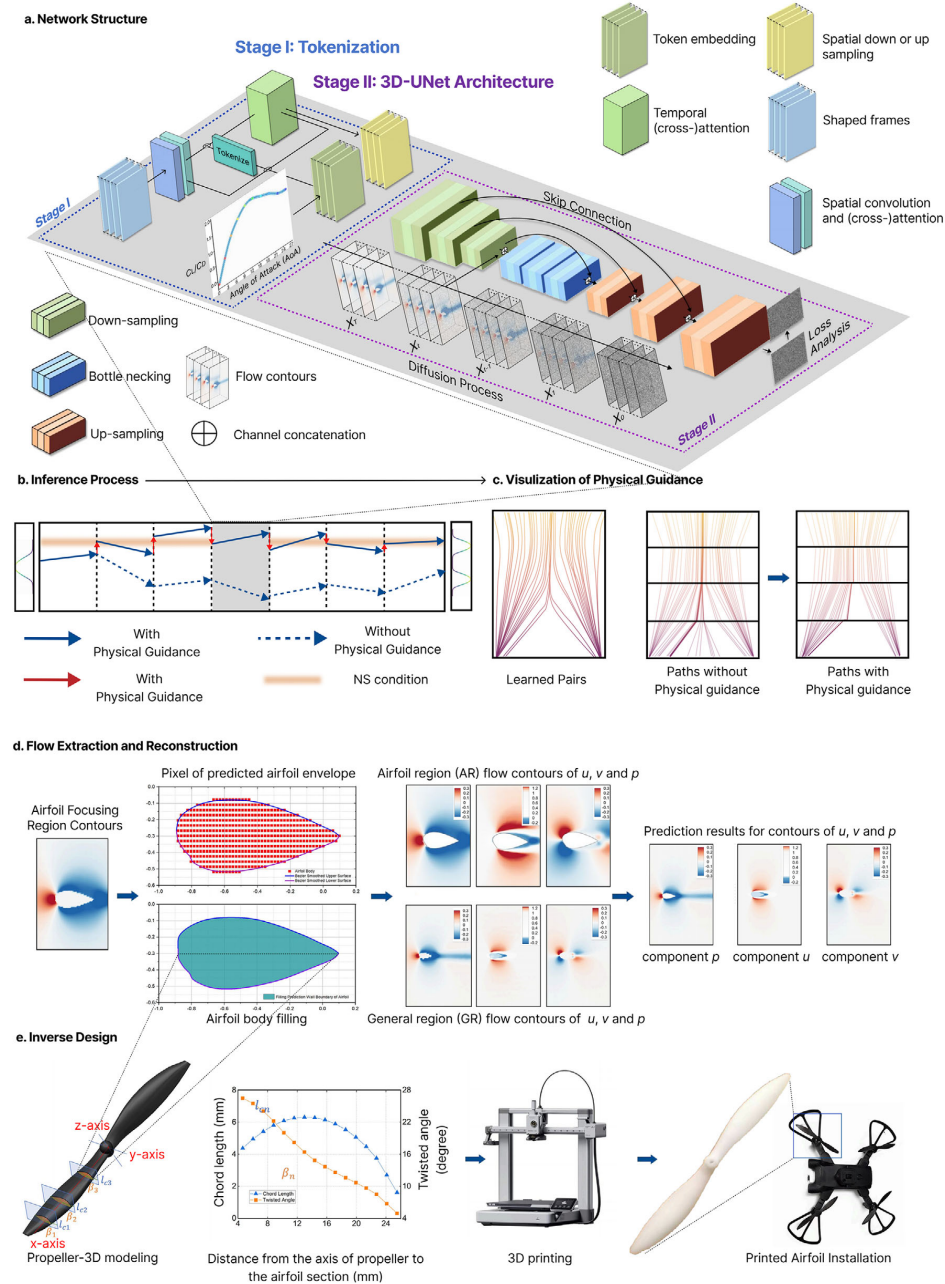
PVDM architecture and merger of full‐field flow contours: (a) Network Structures. Stage I: Tokenization: the *C_L_/C_D_* ratio curves are stored as a vector and then embedded into frames. Blank frames are shaped as: 96 × 96 × 10, which is attached with the spatial convolution and then transferred into the spatial attention. With the tokenized *C_L_/C_D_* values, all of frames based on the increasing rotational angles (AoA) are labeled temporally. Finally, the labeled frames are inserted into the spatial up or down sampling process within the 3D U‐Net of Stage II. Stage II: 3D U‐Net Architecture: the first DDPM illustrated by the purple region reconstructs global flow contour frames via the denoising process, flow fields encompassing both global and local features are generated by averages of frames stochastically sampled from both models; *C_L_*/*C_D_* ratio token sequences are embedded into spatiotemporal convolutional modules via cross‐attention mechanisms; physical guidance is added to restrain the down‐sampling process. (b) Inference Process. The diffusion and denoising processes are displayed along a Markov's chain based on the figure distribution *x_0_*, …, *x_t‐1_*, *x_t_*, *x_T_*, and the physics guidance can be shown as the PDE loss channeled which to minimize the difference between prediction and real interpolated data. (c) Visualization of Physical Guidance. Physical guidance steers diffusion trajectories toward physically consistent manifolds, yielding smoother and divergence‐free streamline evolution. (d) Flow Field Extraction. Airfoil profiles are extracted from flow fields and reconstructed using Bézier‐curve fitting along the edges, finally fused to generate the flow fields of *u*, *v* and *p* and enhance the inverse design. (e) Inverse Design. The output airfoil profiles are modelled in 3D as a propeller and then to be produced by 3D printer, the printed propeller is then installed for further experimental tests.

The variable $z_{\theta }$ is predicted by the U‐Net network, and z is obtained through a denoising process. Drawing inspiration from recent advances in video generative models and inverse design models, our architecture leverages variants of the standard 2D (space‐only) U‐Net, where the applied *C_L_/C_D_* dimension is effectively treated as a batch axis, thereby preserving the integrity of the foundational base network. To ensure physical consistency across varying AoAs, by packaging the velocity and pressure components of the flow field as a three‐channel feature set for network input, we promote a more holistic representation of the flow state. Crucially, this input paradigm provides the necessary basis for introducing physics‐informed regularization during subsequent inference. Two specialized models are trained: one focusing on the airfoil region (AR) and the other on the general flow region (GR).

Figure 2b shows the inference phase of the proposed PVDM. According to Chung et al. [26], an estimate of *x*_0_ can be derived from the following specialized representation of the posterior mean:

(4) $${\hat{x}}_{0}=E\;\left(x_{0}|x_{t}\right)=\frac{1}{\sqrt{{\bar{\alpha }}_{i}}}\;\left(x_{t}+\left(1-{\bar{\alpha }}_{t}\right)\right)\nabla_{x_{t}}\log\left(p_{t}\left(x_{t}\right)\right)$$

Further, the inference result at timestep *t_1_* without correction can be expressed as:

(5)

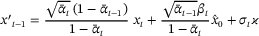

where *σ_t_* = $\sqrt{\beta_{t}}$. It is noted that $\nabla_{x_{t}}\log\left(p_{t}\left(x_{t}\right)\right)$ (and its variants) is always learned by neural networks [27]. The preceding derivation is based on a basic reproduction of the original DDPM formulation, without introducing additional modifications. To add the conditional from the physical ground truth, the data *x* is drawn jointly with conditioning information *c*, such that Equation 4 can be further represented as:

(6) $${\hat{x}}_{0}=\frac{1}{\sqrt{{\bar{\alpha }}_{i}}}\;\left(x_{t}+\left(1-{\bar{\alpha }}_{t}\right)\right)\nabla_{x_{i}}log\left(p_{t}\left(x_{t}\right)|c_{1},c_{2},\text{…},c_{n}\right)$$

Here we make use of the fact that:

(7) $$\begin{array}{ccc} \nabla_{x_{i}}\;\log\left(p_{t}\left(x_{t}\right)|c_{1},c_{2},\text{…},c_{n}\right) & = & \nabla_{x_{i}}\;\log\left(p_{t}\left(x_{t}\right)\right)\\ & & +\sum_{i=0}^{n}\nabla_{x_{i}}log\left(c_{i}|p_{t}\left(x_{t}\right)\right) \end{array}$$

In our case, there are two conditions: lift‐to‐drag ratio guidance *c_i_* and N‐S equation guidance *c_ns_* =  *A*(*x*_0_). Here $\nabla_{x_{t}}log\left(c_{i}|p_{t}\left(x_{t}\right)\right)$ can be obtained by classifier‐free [28] training and sampling. However, $\nabla_{x_{t}}log\left(c_{i}|p_{t}\left(x_{t}\right)\right)$ cannot be learned through the methods above because it is related to *x*_0_. The diffusion posterior sampling (DPS) [29] showed that under the Gaussian noise assumption of the sparse measurement operator *A*(*), the log‐likelihood function can be approximated with:

(8) $$\nabla_{x_{i}}log\left(c_{ns}|p_{t}\left(x_{t}\right)\right)\approx ∥A\left(x_{0}\right)-A\left({\hat{x}}_{0}\right){∥}^{2},$$

where $\hat{x}$ *_0_* is an estimated value of *x_0_* through Equation 4. Given the dimensionless, incompressible, steady Reynolds‐averaged Navier–Stokes governing equations for flow:

(9) $$\nabla \cdot \bar{u}=0$$

(10) $$-\bar{u}\cdot \nabla \bar{u}-\nabla \bar{p}+\frac{1}{Re}\nabla^{2}\;\bar{u}=R$$

where 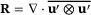 represents Reynolds‐stress output from DNS, $\bar{u}$ is the mean velocity field ($\bar{u}=(\bar{u},\;\bar{v})$). Hence, we have the N‐S physical correction (hyperparameter) $L_{ns}$ as:

(11) $$L_{ns}=∥0-f\left({\hat{x}}_{0}\right)∥\;$$

and:

(12)

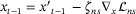

where *x*′_*t* − 1_ is the result of *C_L_*/*C_D_* guidance according to Equation 5, *f*(*) represents the N‐S function, and $L_{ns}$ is the N‐S constraint coefficient.

We provide 3 pages for further details of the derivation of the PVDM in Section S2), where the algorithm is given. By integrating N‐S constraints, we substantially mitigate the epistemic uncertainty inherent in diffusion models. This yields reconstructed wing geometries and synthesized flow fields that demonstrate enhanced adherence to fundamental physical laws.

Figure 2d illustrates the complete workflow from flow‐field reconstruction to geometry extraction and experimental validation. The rigid‐wall boundaries are first identified from the ground‐truth fields as pixels satisfying (*u*,  *v*,  *p*  =  0). For each streamwise location, the maximum and minimum *y*‐coordinates of the boundary pixels are extracted to define the upper and lower airfoil surfaces, respectively. These discrete surface coordinates are subsequently connected and smoothed using Bézier curves to reconstruct a continuous airfoil profile. The reconstructed airfoil geometry is then used to construct the propeller blade by progressively rotating and positioning the sectional profiles along the *x*‐axis. The resulting propeller geometries are fabricated by three‐dimensional printing and experimentally evaluated on unmanned aerial vehicles to assess their flight performance. As representative demonstrations, two generated airfoils, NACA5190 and NACA2145, are integrated into the propellers and tested under practical flight conditions. The corresponding experimental demonstrations are provided in Video S2. In addition, the training file formats, hyperparameters, and corresponding platform information details, respectively are shown in Tables S1–S3.

### 2.3 High‐Fidelity CFD Simulation via the High Spectral Element Method and *C_L_/C_D_* Response Extraction

Here we use a high‐order spectral/hp element discretization [30], which expands the solution with degree‐*p* polynomials on each element of an unstructured mesh, combining the geometric flexibility of finite elements with the resolution properties of spectral methods. The formulation supports *h*‐ (mesh) and *p*‐ (polynomial) refinement, as well as their *hp* combination, yielding algebraic error decay *O*(*h^P^*) or sufficiently smooth fields. We adopt a standard Galerkin weak form with *C* ^0^‐continuous expansions and element‐level operators, which enables efficient solvers (e.g., static condensation) while preserving high‐order accuracy. In the present study, the polynomial order is set as 4 in the global domain, which is much higher than the conventional finite volume method (second order). Along with DNS simulation, we can obtain the high‐fidelity flow field. Here, we use a semi‐implicit scheme to directly solve the N‐S equations shown in Equations (9) and (10). The scheme discretizes the momentum Equations (9 and 10) with a backwards approximation of the time derivative to obtain:

(13) $$\frac{\partial u^{n+1}}{\partial \tau }≃\frac{\gamma_{0}{\tilde{u}}^{n+1}-\hat{u}}{\Delta \tau }$$

where *u* ^*n* + 1^ is an intermediate velocity and $\hat{u}$ is the summation of previous solutions. With the discrete time derivative, we initially solve a pressure Poisson equation of the form:

(14) $$\nabla^{2}\;p^{n+1}=\nabla \cdot \left(\frac{\hat{u}}{\Delta \tau }-N^{*,n+1}\right)$$

and use the consistent Neumann boundary conditions prescribed as:

(15) $$\frac{\partial p^{n+1}}{\partial n}=\;-\left[\frac{\partial u^{n+1}}{\partial \tau }+\nu {\left(\nabla \times \nabla \times u\right)}^{*,n+1}+N^{*,n+1}\right]\cdot n$$

The advection term is $N^{*,n+1}={\left[u\cdot \nabla u\right]}^{*,n+1}$, where the superscript indicates extrapolation from previous solutions.

Subsequently, we solve a Helmholtz equation for new velocity [*u* ^n + 1^ *, v* ^n + 1^, *w* ^n + 1^], thus:

(16) $$\left(\Delta -\frac{\gamma_{0}}{\nu \Delta \tau }\right)\;u^{n+1}=-\left(\frac{\gamma_{0}}{\nu \Delta \tau }\right)\hat{u}+\frac{1}{\nu }\nabla p^{n+1}$$

Generally, this scheme solves for the new pressure *p* ^n + 1^ and velocity *u* ^n + 1^ based on initial conditions at τ^n^ =  *n*Δτ and boundary conditions. The algorithm proceeds in three steps: first, the advection terms are evaluated; second, a Poisson problem for the updated pressure *p* ^n + 1^ is solved; finally, a Helmholtz problem is solved for each velocity component.

The validation details of lift and drag coefficients are shown in Section S3.3 at *p*  =  3,  4,  5,  6. A *p*‐th order of *p*  =  4 is selected for all general flow domain calculation cases to balance computational accuracy with time and storage costs. It should be mentioned that all physical variables are dimensionless.

The lift and drag force coefficients (*C_L_*/*C_D_*) are extracted from the temporal snapshots involving full‐field *u*, *v*, and *p* distribution data over time, as follows:

(17) $$C_{L}=2F_{L}/\rho U^{2}L$$

(18) $$C_{D}=2F_{D}/\rho U^{2}L$$

Here, the working fluid density ρ is assumed to be 1 kg/m^3^, *F_L_* is the applied vertical lift aerodynamic force on the airfoil, and *F_D_* is the horizontal drag aerodynamic force applied on the whole airfoil.

### 2.4 Normalization of DNS Data

All discretized 2D Reynolds‐average full‐field flow data (*u*, *v*, and *p*) and *C_L_*/*C_D_* ratio tokens obtained from the DNS process were normalized according to the following process based on their respective global maximum and minimum values obtained over all matrix coordinates, denoted as *max*(*V_global_*) and *min*(*V_global_*), respectively.

(19) $$\begin{array}{c} V_{normalized}\left(idx,idy\right)=\frac{V_{orginal}\left(idx,idy\right)-min\left(V_{global}\right)}{max\left(V_{global}\right)-min\left(V_{global}\right)+10^{-5}} \end{array}$$

Here, the position of each pixel value is denoted as (*idx*,  *idy*). The global maximum and minimum values of the flow data are obtained as *max*(*V_global_*)and *min*(*V_global_*). Therefore, all normalized data have been restrained within the range [−1, 1], while a constant “10^−5^” is included in the denominator to avoid division by zero under the unlikely case where *max*(*V_global_*)  =  *min*(*V_global_*).

### 2.5 2D U‐Net Framework

Herein, we summarize the spatial 2D U‐Net framework [31] at a conceptual level. More comprehensive technical implementation details can be referred to ‘Code Availability’. All implementations apply the PyTorch framework (*v*2.1+) [32]. Because diffusion models operate through iterative noise removal from typically image data, the U‐Net architecture must meet the requirement for identical input‐output dimensions. Our model extends the foundational 2D U‐Net architecture to accommodate the video diffusion framework and its reference implementation [33]. The applied encoder‐decoder structure progressively compresses spatial information while expanding latent features (down‐sampling), and then inversely reconstructs spatial resolution from latent representations (up‐sampling). Each downsampling/ upsampling stage integrates the following features:

- dual residual neural network (ResNet) blocks with convolutional layers and sigmoid linear unit (SiLU) activation functions σ(*x*)  =  *x* · *sigmoid*(β*x*) [34];
- spatially factorized self‐attention for enhanced computational efficiency [35];
- strided convolutions for resolution scaling.

The bridge module acting between encoder and decoder similarly employs dual ResNet blocks with full self‐attention interleaved [36]. We deployed four hierarchical resolutions (96 × 96→12 × 12) with exponentially increasing latent dimensions (64→512), where each multi‐head attention block contains 8 heads (dimension = 32 per head). The critical hyperparameters employed during network training are cataloged in Section S3.2 along with details regarding the experimental platforms employed for dataset generation, model training, and model testing.

### 2.6 Extension to Temporal 3D U‐Net Architecture

The foundational 2D U‐Net architecture was extended into a temporal 3D architecture according to a previously reported process [37] that integrated a quasi‐temporal dimension representing an applied mechanical process of airfoil swinging associated with AoAs, and treated this dimension as a batched axis to maintain structural consistency across all building blocks. This architecture is clearly illustrated schematically in Figure 2, where, crucially, a temporal self‐attention layer is embedded at a point prior to the encoder‐decoder framework and iteratively after each spatial attention module, with spatial axes batched to enable attention across 10 discrete *C_L_*/*C_D_* intervals. In addition, a previously reported relative positional encoding *P_rel_* (*t_i_*, *t_j_*) is implemented at times *t_i_* and *t_j_* to encode AoA‐sequence dependencies. This encoding thereby preserves the ordering of the angle‐of‐attack frames, thus allowing the diffusion model to capture variations in the mean flow across incidence angles, as demonstrated in stage II of Figures 3a.

**FIGURE 3:**
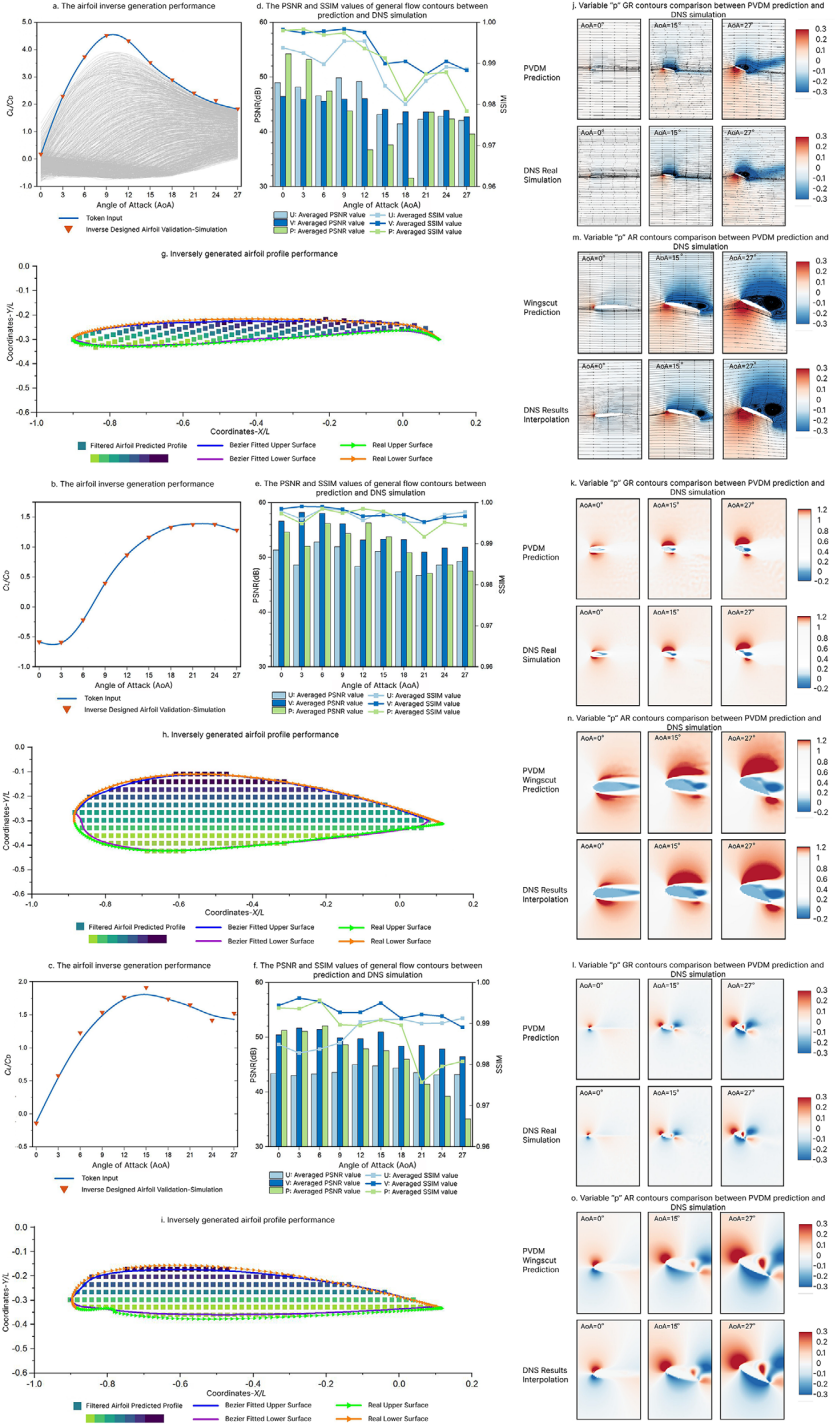
Inverse airfoil prediction and full‐field flow reconstruction results for representative NACA airfoils: NACA5190, NACA5431, NACA7122. (a–c) *C_L_*/*C_D_* response at all angles of attack (AoA) for NACA 5910, 5431 and 7122, respectively. d‐f, PSNR, SSIM values of general flow contours between prediction and DNS simulation. (g–i) Actual and inversely generated airfoil profiles from Bezier curve fitting. (j–l) Comparison of predicted and simulated velocity and pressure distributions obtained for selected AoA values with 0°, 15° and 27° at 96 × 96 pixels global regions. (m–o) Comparison of predicted and simulated velocity and pressure distributions obtained for selected AoA values with 0°, 15° and 27° at 256 × 256 pixels global regions.

**FIGURE 4:**
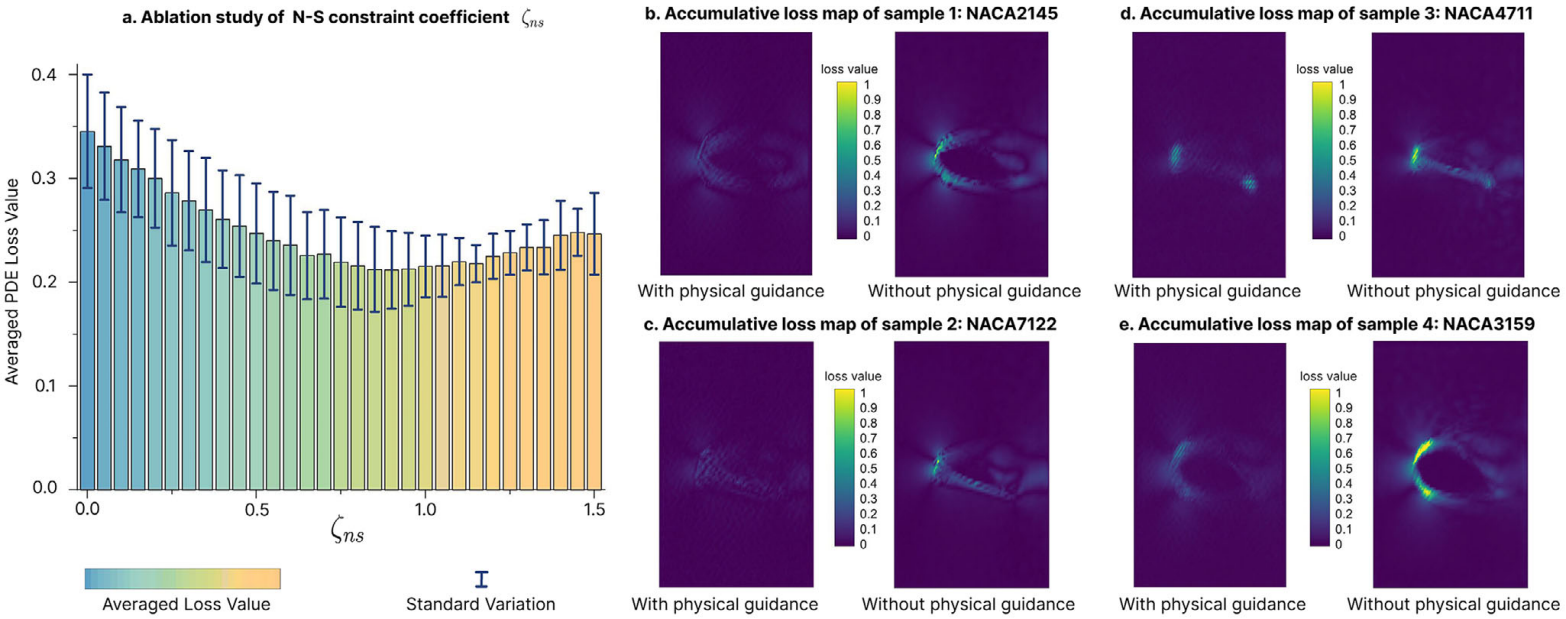
Evaluation of physical consistency and hyperparameter sensitivity. (a) Average PDE loss (mean ± s.d.) across all sampling cases as a function of the loss function hyperparameter, identifying an optimal weighting that minimizes physical residuals. (b–e) Cumulative PDE loss maps for the NACA2145 (b), NACA7122 (c), NACA5431 (d), NACA3159 (e) airfoils. Both panels contrast the prediction with physical guidance (left) against the unguided baseline (right), visualizing normalized per‐pixel residuals (scaled × 10).

### 2.7 Conditioning PVDM on *C_L_*/*C_D_* Response to AoA

The PVDM was conditioned on the *C_L_*/*C_D_* response to AoA by projecting all of 10 AoA values into a latent embedding via a parameterized linear projection $W\in R^{d\times 10}$. These embeddings are concatenated to spatial attention tokens for cross‐attention, where queries derive from pixel embeddings and keys/values derive from conditioning embeddings, while temporal attention tokens integrate all 10 embeddings with the relative positional encoding *P_rel_* (*t_i_, t_j_*) [38] discussed in the preceding subsection to preserve mechanical chronology. Crucially, a conditioning amplifier averages AoA‐step embeddings and transforms them via a two‐layer multilayer perceptron (MLP) with SiLU activations (*β* = 1.702), which projects outputs to match the dimensionality of the latent space of diffusion timestep *t*. This fused representation $z_{t}⊕z_{c}$ is additively incorporated into the ResNet blocks of the PVDM, which synchronizes denoising dynamics with aerodynamic conditioning, while suppressing spectral leakage artifacts, as demonstrated in Figures 3a.

## 3 Results and Discussion

### 3.1 Full‐Field Flow Predictions

The generalization performance of the trained PVDM model in predicting the *C_L_*/*C_D_* response beyond the training protocol was evaluated using a testing dataset partitioned into three distinct test cohorts: (i) high *C_L_*/*C_D_* magnitude variants (1/3 of all samples), where curves is generally above the top curve of training data (NACA 5810 airfoil: 330033, 2.272060, 3.659395, 4.385132, 4.247737, 3.528141, 2.782431, 2.322401, 2.048663, 1.769083); (ii) profiles with curvature slopes exceeding all training samples (1/3 of all samples), where curve's slope is generally above the largest curve of training data (NACA 4910 airfoil's slope: 0.685333, 0.573000, 0.352167, 0.081833, −0.148500, −0.225833, −0.179833, −0.113000, −0.077333, −0.053667); (iii) geometrically analogous samples (the remaining 1/3), where A ≥ 6, B ≥ 1, C ≥ 10. Three representative airfoils are demonstrated in Figure 3: NACA5910 in test cohort (i), NACA5431 in test cohort (ii), and NACA7122 in test cohort (iii).

The PVDM demonstrates robust inverse design capabilities. Utilizing the target *C_L_*/*C_D_* trajectories obtained from DNS as input (Figure 3a–c, bold blue lines), the model accurately reconstructs the corresponding airfoil profiles. The accuracy performance of this geometric reconstruction is evidenced in Figure 3g–i, where the Bezier‐fitted predictions exhibit exceptional agreement with the ground‐truth theoretical envelopes. To validate the aerodynamic performance of these inversely generated geometries, we performed DNS re‐simulations on the predicted profiles. The resulting *C_L_*/*C_D_* response (Figure 3a–c, orange scatter points) align precisely with the target inputs, confirming the functional accuracy of the design.

Beyond scalar airfoil profile, the capacity of the model to predict full‐field flow variables was assessed. A comparison of the velocity (*u*, *v*) and pressure (*p*) distributions (Figure 3j–o) reveals that the PVDM‐predicted fields are qualitatively indistinguishable from the DNS baselines across varying AoAs. This visual consistency is substantiated quantitatively by high Peak Signal‐to‐Noise Ratios (PSNR, 34.20–54.35 *dB*) and Structural Similarity Index Measures (SSIM > 0.8) as shown in Figure 3d–f, which also present an averaged fidelity larger than 90% based on the high SSIM results referring to Table S5. These metrics confirm that the proposed approach achieves high‐fidelity prediction of 2D flow fields solely through PVDM inference, bypassing the computational cost of traditional solvers. Comprehensive results across all 10 AoA values are detailed in Section S5. To further assess the generalizability of the model, additional prediction examples including all of 10 AoA frames are provided in Figures S5–S12, and inversely generated airfoil profiles can be further displayed by S13 and S14. A video referring to Video S1 displays 4 samples for comparing prediction and DNS results with a mechanically changing AoA process.

Beyond statistical similarity metrics, we scrutinized the physical validity of the generated flow fields by analyzing the residuals of the governing equations. Figure 4 summarizes the PDE loss characteristics, highlighting the impact of the physics‐informed constraints. The cumulative loss maps presented in Figure 4a,b visualize the normalized per‐pixel deviations aggregated over the *u*, *v*, and *p* components for the NACA5431 and NACA7122 airfoils.

A comparative assessment reveals that the inclusion of physical guidance markedly suppresses spatial errors and high‐frequency artifacts relative to the unguided baseline. This mitigation confirms that explicitly enforcing incompressible Navier–Stokes constraints (Equations 9 and 10) during inference effectively regularizes the flow field prediction. Furthermore, the sensitivity of the model to the loss‐weighting hyperparameter is quantified in Figure 4a. The optimization landscape demonstrates a convex profile, attaining a global minimum PDE loss of 0.212 (± 0.037 s.d.) at the optimal hyperparameter setting. This indicates a stable operating regime where the balance between data fidelity and physical consistency is maximized. The cumulative PDE loss maps of 4 samples (both NACA2145 and NACA7122 are shown in the results, and both NACA4711 and NACA3159 are the other randomly selected samples) are displayed in Figure 4b–e, where the color map indicates the absolute value difference between prediction and DNS simulation.

### 3.2 Airfoil Profile Inverse Design

The wall boundary location was estimated by identifying flow field pixels where the absolute values of pressure |*p*|, streamwise |*u*|, and spanwise |*v*| velocity components simultaneously fell within the interval 0.01< {|*p*|, |*u*|, |*v*|} < 0.01. These locations were recorded by their corresponding column and row indices. As illustrated in Figure 2d, the airfoil body was subsequently reconstructed as the total area enclosed by fitted Bezier curves derived from this boundary data. To minimize boundary edge error, which is concentrated in the wall boundary layer region (Figure 4b,c), the results from 10 independent samples (comprising *u*, *v*, and *p* fields) were averaged. The extracted profiles, initially obtained over 10 different AoAs, were then corrected back to the zero‐rotation angle (0°). Final calibration was performed by calculating the average y‐coordinate values at each x‐coordinate for both the upper and lower surfaces. The final performance of the airfoil profile reconstruction is summarized in Figure 3g–i.

Utilizing the reconstructed airfoil profile, a propeller model was designed across the radial distance from 4 to 26 mm. The cross‐sectional twisted angle evolved non‐linearly, decreasing gradually from 26° to 5°. Concurrently, the chord length showed a non‐monotonic change, increasing from 4.3 to 6.3 mm and then decreasing to 1.8 mm. The resulting 3D model was fabricated (3D printed) and installed on the test rig for the subsequent flying test, as documented in Video S2.

### 3.3 Discussion

The proposed physics‐informed video diffusion model (PVDM) achieves efficient inverse airfoil design by relying solely on compact lift‐to‐drag ratio tokens. Each prediction requires approximately 30 s of computation, compared with the several hours typically needed for iterative optimization based on conventional CFD simulations. This accelerated framework demonstrates substantial potential for mission‐specific airfoil tailoring in unmanned aerial vehicles (UAVs), enabling rapid adaptation to low‐altitude flight regimes relevant to delivery, surveillance, and inspection tasks. The model accurately reconstructs low‐Reynolds‐number flow fields and provides dynamic predictions of velocity and pressure distributions across varying angles of attack, with a mean contour error below 5%. The proposed dual‐model strategy mitigates resolution‐dependent artifacts, while the PVDM reduces the effective computational cost of flow‐field prediction by nearly 600‐fold compared with DNS‐based simulations. In addition, the generated NACA–Nek1000 dataset provides publicly accessible, high‐precision flow data at *Re* = 1000 for micro‐aerial robot research. Collectively, these contributions establish a foundation for rapid, physics‐informed aerodynamic design and flow visualization under low‐Reynolds‐number conditions.

The PVDM still exhibits limited generalization when exposed to distributionally shifted *C_L_*/*C_D_* tokens, which constrains its extrapolative capability. High‐resolution training at or above 256 × 256 can introduce duplication or ghosting artifacts in predicted contours. These artifacts may be attributed to a combination of limited long‐range dependency modeling in the U‐Net backbone, noise amplification in sparsely sampled regions, and discontinuities near airfoil‐wall interfaces during block‐wise processing. Moreover, the current model is trained exclusively on low‐Reynolds‐number laminar flow conditions at *Re* = 1000, limiting its direct applicability to transitional or turbulent regimes. Although diffusion transformers (DiTs) offer stronger global feature extraction and improved spatial coherence, their substantial computational demands and data requirements currently hinder their deployment for high‐resolution aerodynamic modeling.

## 4 Conclusions and Implications

This work supports a shift toward generative, physics‐informed frameworks in which aerodynamic design and flow prediction are no longer tightly coupled to complex meshing procedures or iterative numerical solvers. By introducing a lift‐to‐drag ratio‐conditioned generative process, the proposed framework reframes inverse aerodynamic design as a direct mapping problem and provides a rapid optimization feedback mechanism for near‐real‐time aerodynamic reasoning. In doing so, it outlines a new direction for integrating generative AI with physics‐informed modeling, where aerodynamic analysis can become more accessible, efficient, and adaptable.

Future progress will depend on expanding both the physical and statistical coverage of the model. Incorporating datasets across broader Reynolds number ranges, flow regimes, and geometric configurations would enable more reliable generalization across laminar, transitional, and turbulent flows. In parallel, physically grounded token representations, such as characteristic flow features or tokenized performance curves, could be embedded into the diffusion process to improve consistency with governing equations, including the Navier–Stokes equations. At the architectural level, integrating global context mechanisms or evolving the framework toward more efficient Diffusion Transformer architectures may further enhance coherence, controllability, and the ability to reason directly from textual, symbolic, or high‐level aerodynamic design specifications.

Looking beyond inverse design, this framework could be extended to three‐dimensional wing design and complex aerodynamic structures, where geometric constraints, multi‐objective performance demands, and flow–structure interactions make conventional optimization expensive and time‐consuming. As lightweight physics‐informed surrogates, generative models may accelerate the exploration of high‐dimensional design spaces while preserving mechanically meaningful quantities such as pressure distributions, force responses, separation behavior, and stability indicators. The same paradigm may further benefit experimental fluid mechanics, particularly wind‐tunnel measurements based on particle image velocimetry, where data acquisition is often limited by optical access, seeding quality, sampling frequency, and instrumentation cost. Physics‐informed generative models could reconstruct globally coherent flow fields from sparse PIV observations, reducing the need for dense experimental acquisition while retaining interpretable vortical structures and dynamically consistent flow evolution. In the longer term, coupling diffusion or flow matching with explicit physical constraints may enable real‐time generation of physically consistent flow fields from sparse sensor, visual, or symbolic inputs. Such capabilities could support vision‐language‐action systems in embodied intelligence, allowing agents to reason about aerodynamic affordances, fluid–structure interactions, and dynamic environmental feedback. Together, these directions suggest that generative modeling may reduce both the computational cost of aerodynamic optimization and the experimental cost of acquiring high‐fidelity flow data, expanding fluid‐dynamic intelligence across design, measurement, and autonomous decision‐making.

## Author Contributions

**Yifeng Ai**: conceptualization, methodology, software, validation, formal analysis, investigation, data curation, writing – original draft, visualization. **Yijia Guo**: conceptualization, methodology, software, validation, investigation, data curation, formal analysis, visualization, writing – original draft. **Hongfu Zhang**: conceptualization, methodology, writing – original draft, data curation, validation. **Lei Zhou**: conceptualization, investigation, writing – review and editing, data curation. **Tiejun Huang**: supervision. **Weiqiu Chen**: writing – review and editing. **Xuhui He**: supervision. **Peng Guo**: investigation, data curation. **Lei Ma**: supervision, project administration, writing – review and editing, funding acquisition, resources.

## Funding

The work was supported by National Natural Science Foundation of China (Project No. 12572031) and Beijing Natural Science Foundation (JQ24023).

## Conflicts of Interest

The authors declare no conflicts of interest.

## Supporting information




**Supporting File 1**: advs78532‐sup‐0001‐SuppMat.docx.

**Supporting File 2**: advs78532‐sup‐0002‐VideoS1.mp4.

**Supporting File 3**: advs78532‐sup‐0003‐VideoS2.mp4.

## Data Availability

The training and validation protocol, including the token response and corresponding full‐field flow data distributions (variables *u*, *v*, and *p*) for the DNS‐solved global region and airfoil‐focused region calculation subdomains is posted at https://huggingface.co/datasets/diff‐flow/diff‐flow/tree/main, where the pretrained PVDMs are given in https://huggingface.co/diff‐flow/diff‐flow/tree/main. The post data processing code and the main code used for training the network applied for mapping connections between *C_L_*/*C_D_* ratio response and corresponding frames of full‐field *u*, *v*, and *p* distributions is available at https://github.com/insightlab‐CG‐3DV/diffusion‐model‐of‐inversely‐design‐airfoil‐flowcontours.
