# Supplementary material for: Accelerating High‐Fidelity Airfoil Design via Physics‐Informed Video Diffusion Model
Source: Adv Sci (Weinh). 2026 Nov 3:e78532. Online ahead of print. doi: 10.1002/advs.78532 (PMC13731080; doi:10.1002/advs.78532)
Supplement: Supplementary file 1 [file ADVS-9999-e78532-s003.docx]

# **Supporting Information for “Accelerating High-fidelity Airfoil Design via Physics-informed Video Diffusion Model”**

**This supporting information includes:**

Supporting text

Figures S1 to S14

Tables S1 to S5

**Other supporting information for this manuscript include the following:**

Movie S1 to S2

# **Supplementary Information Text**

## 1. **Generation of full-field flow data (u, v, and p)**

### 1.1. **Definition of the airfoil profile**

The two-dimensional (2D) airfoil profile definition process employed herein based on the 4-digit NACA code involving parameters *A*, *B*, and *C* defines the camber line with respect to the *x* direction according to the following equations:

| $y_{c}=\frac{A}{\left( B*0.1 \right)^{2}}(0.2Bx-x^{2})$ when $x<0.1B$ | (1) |
| --- | --- |
| $y_{c}=\frac{A}{\left( 1-B*0.1 \right)^{2}}\left( 1-0.2B+0.2Bx-x^{2} \right)$ when $x\geq0.1B$ | (2) |

Where the *y_c_* denotes the y-coordinates of the points located at the camber line. The thickness (*C*%) distribution *y_t_* is given by the following equation:

$$y_{t}=0.05C(0.2969x^{0.5}-0.1260x-0.3516x^{2}+0.2843x^{3}-0.1015x^{4})$$

(3)

The angle *θ* between the line tangent to the camber line and the horizontal *x*-axis is presented as:

$$\theta=atan(\frac{dy_{c}}{dx})$$

(4)

Then, the coordinates of the upper surface of the airfoil can be expressed as follows.

$$x_{u}=x_{c}-y_{t}sin(\theta)$$

(5)

$$y_{u}=y_{c}+x_{t}cos(\theta)$$

(6)

Similarly, the lower surface coordinates can be expressed as follows.

$$x_{l}=x_{c}+y_{t}sin(\theta)$$

(7)

$$y_{l}=y_{c}-x_{t}cos(\theta)$$

(8)

### 1.2. **Direct numerical simulations**

The direct numerical simulation (DNS) approach applied herein to obtain the 2D incompressible full-field flow solutions with high accuracy involves direct solutions of the following Navier-Stokes equations, which has been shown in Equation 9 and 10 of the main text. Here, *u* is the flow velocity, which can be either the stream-wise flow speed in the *x* direction or the span-wise flow speed *v* in the *y* direction, *p* is the pressure, *Re* represents the Reynolds number, and $\tau$ is time.

| ∇$\cdot$u = 0 | (9) |
| --- | --- |
| $\frac{\partial u}{\partial\tau}=-u\cdot\nabla u-\nabla p+\frac{1}{Re}\nabla^{2}u$ | (10) |

## 2. **Physical-guided video denoising diffusion probabilistic model**

Here, we provide a detailed review of the formulation of Gaussian diffusion models according to DDPM. The forward diffusion process is defined by a fixed Markov chain with given variance schedule *β_t_*.

$$q\left( x_{t} | x_{t-1} \right)\mathcal{=N}\left( x_{t};\sqrt{1-\beta_{t}}x_{t},\beta_{t}I \right), t\in\{0,1,\ldots,T-1\}$$

(11)

where the $x_{1}, ...,x_{T}$ are latents of the same dimensionality as the data $x_{0}\sim q(x_{0})$, *β*∈ (0,1) is the scheduled variance and *t* is the timestep, $\mathcal{N}$ denotes a Gaussian distribution. We need not apply *q* repeatedly to sample from $x_{t}\sim q({x_{t}|x}_{0})$. Instead, it can be represented as a Gaussian distribution:

$$q\left( x_{t} | x_{t-1} \right)\mathcal{=N}\left( x_{t};\sqrt{\bar{\alpha}_{t}}x_{t},{(1-\bar{\alpha}}_{t})I \right)=\sqrt{\bar{\alpha}_{t}}x_{0}+\sqrt{1-\bar{\alpha}_{t}}z;z\mathcal{\sim N}(0,1)$$

(12)

where:

$$\alpha_{t}=1-\beta_{t},\bar{\alpha}_{t}=\prod_{i=0}^{t} (1-\alpha_{t})$$

(13)

According to the Bayes theorem, the posterior *q*(*x_t_*|*x_t-1_, x_0_*) also a Gaussian with mean $\hat{\mu}$*_t_*(*x_t_, x_0_*) and variance$\hat{\beta}$*_t_* is defined as follows:

$$\mu_{t}\left( x_{t},x_{0} \right)=\frac{\sqrt{\bar{\alpha}_{t-1}}\beta_{t}}{1-\alpha_{t}}x_{0}+\frac{\sqrt{\bar{\alpha}_{t}}{(1-\alpha}_{t-1})}{1-\alpha_{t}}x_{t}$$

(14)

$$\hat{\beta}_{t}=\frac{1-\bar{\alpha}_{t-1}}{1-\bar{\alpha}_{t}}\beta_{t}$$

(15)

$$q\left( x_{t} | x_{t-1} \right)=N\left( x_{t-1};\hat{\mu}_{t}(x_{t},x_{0}),\hat{\beta}_{t}I \right)$$

(16)

If we want to have a sample from the target unknown distribution *q*(*x_0_*), we can directly sample from an isotropic Gaussian distribution *q*(*x_T_*) and reversely sample *q*(*x_t_*|*x_t-1_*). We can use neural networks to approximate the unknown *q*(*x_t_*|*x_t-1_*). Considering:

$$p_{\theta}\left( x_{t} | x_{t-1} \right)=N\left( x_{t};\mu_{\theta}(x_{t},t),\sum_{\theta}(x_{t},t) \right)$$

(17)

To train this model, we can optimize the variational lower-bound for *p_θ_*(*x_0_*). In actual training, we simplify the optimization objective as:

$$L_{simple}=E_{t\sim\left[ 1,T \right],x_{0}\sim q\left( x_{0} \right),z\sim N(0,1)}[\left\| z-z_{\theta}(x_{0},t) \right\|]$$

(18)

The former is an unconditional reverse-noising process. In the case of conditional generative modeling with condition *c*, we have:

$$p_{\theta,\phi}\left( x_{t} | x_{t+1},c \right)=Kp_{\theta}(x_{t}|x_{t+1})p_{\phi}(c|x_{t})$$

(19)

By applying

$$\log{(p}_{\theta}\left( x_{t} | x_{t+1} \right))=-\frac{1}{2}\left( x_{t}-\mu\right)^{T}\sum^{-1}\left( x_{t}-\mu\right)+C$$

(20)

and

$$\log{(p}_{\phi}\left( c | x_{t} \right))\approx\left( x_{t}-\mu\right)g+C_{1}$$

(21)

Here $g={\nabla_{x_{t}}\log{(p}_{\phi}\left( c | x_{t} \right)|}_{x_{t}=\mu})$, C and C_1_ are constant. This gives:

$$\log(p_{\theta,\phi}\left( x_{t} | x_{t+1},c \right))\approx\log(p\left( z \right))+C_{2},z\sim N(\mu+\sum g,\sum)$$

(22)

We have thus found that the conditional transition operator can be approximated by a Gaussian similar to the unconditional transition operator, but with its mean shifted by Ʃ*g*. For the forward process, if we have a model *є*_θ_(*x_t_*) that predicts the noise added to a sample, then this can be used to derive a score function:

$$\epsilon_{\theta}\left( x_{t} \right)=\frac{1}{\sqrt{1-\bar{\alpha}_{t}}}\nabla_{x_{t}}log(p_{t}(x_{t}))$$

(23)

Substituting this into the score function for *p_θ_*(*x_t_*)*p_ϕ_*(*c*|*x_t_*), we have:

$$\nabla_{x_{t}}\log(p_{\theta}\left( x_{t} \right)p_{\phi}\left( c | x_{t} \right))=\frac{1}{\sqrt{1-\bar{\alpha}_{t}}}\varepsilon_{\theta}\left( x_{t} \right)+\nabla_{x_{t}}log(p_{\phi}c|x_{t})$$

(24)

Finally, we can define a new epsilon prediction $\hat{\epsilon}$*_θ_*(*x_t_*) which corresponds to the score of the joint distribution, as:

$$\hat{\epsilon}_{\theta}\left( x_{t} \right)=\epsilon_{\theta}\left( x_{t} \right)-\sqrt{1-\bar{\alpha}_{t}}\nabla_{x_{t}}log(p_{\phi}(c|x_{t}))$$

(25)

In this paper, the condition *c* is the *C_L_*/*C_D_* guidance. The above derivation for conditional sampling is only valid for the training stage. For the testing stage, we take PDE guidance into account:

$$p_{\theta,\phi}\left( x_{t} | x_{t+1},c,y \right)=Kp_{\theta}(x_{t}|x_{t+1})p_{\phi}(c|x_{t})p_{\phi}(y|x_{t})$$

(26)

where $y=A(x0)+n$ and $A(*)$ represent the PDE guidance. Further we have:

$$\nabla_{x_{t}}\log(p_{\theta}\left( x_{t} | c,y \right))=\nabla_{x_{t}}\log\left( p_{\theta}\left( x_{t} \right) \right)+\nabla_{x_{t}}\log(p_{\phi}\left( c|x_{t} \right))+\nabla_{x_{t}}\log(p_{\phi}\left( y|x_{t} \right))$$

$$=\hat{s}_{\theta}\left( x_{t},c \right)+\nabla_{x_{t}}log(p_{\phi}\left( y|x_{t} \right))$$

(27)

According to Equation 25, $\hat{s}_{\theta}\left( x_{t},c \right)=\nabla_{x_{t}}\log(p_{\theta}\left( x_{t} \right))+\nabla_{x_{t}}\log(p_{\phi}\left( c|x_{t} \right))$ can be computed by $\epsilon_{\theta}(x_{t})$ which is predicted by a learned model. We just need to estimate $\nabla_{x_{t}}\log(p_{\phi}\left( y|x_{t} \right))$*.* From Chung et al., 2023, for the given measurement model with $n\sim\mathcal{N}(0,\sum I)$, we have:

$$p_{\phi}\left( y|x_{t} \right)\approx p_{\phi}(y|\hat{x}_{0})$$

(28)

By leveraging the result of equation (28), we have:

$$\nabla_{x_{t}}\log(p_{\phi}\left( y|x_{t} \right))\approx-\frac{1}{\sum}\nabla_{x_{t}}\left\| A\left( x_{0} \right)-A(\hat{x}_{0}(x_{t})) \right\|_{2}^{2}$$

(29)

Plugging in the result from equation (27), we have:

$$\nabla_{x_{t}}\log(p_{\theta,\phi}\left( x_{t} | c,y \right))\approx\hat{s}_{\theta}\left( x_{t},c \right)-\rho\nabla_{x_{t}}\left\| y-A(\hat{x}_{0}(x_{t})) \right\|_{2}^{2}$$

(30)

Given the governing equations for flow:

| ∇$\cdot\bar{u}$ = 0 | (31) |
| --- | --- |
| $-\bar{u}\cdot\nabla\bar{u}-\nabla\bar{p}+\frac{1}{\mathrm{Re}}\nabla^{2}\bar{u}=R$ | (32) |

Here $\text{x}$ denote the predicted physical field. The superscript indicates the field type: i.e., $\text{x}^{\bar{\text{u}}}$ and $\text{x}^{\bar{\text{p}}}\text{ }$denote the mean velocity field $\text{x}^{\bar{\text{u}}}\text{=(}\bar{\text{u}}\text{, }\bar{\text{v}}\text{)}$ and mean pressure field, respectively. $\text{R}\text{=}\text{∇}\text{∙}\bar{\text{u}\text{'}\text{⨂}\text{u}\text{'}}$ represents Reynolds-stress calculated from DNS. Hence, we have:

$$\nabla_{x_{t}}\log\left( p_{\phi}\left( y | x_{t} \right) \right)\approx-\frac{1}{\sum}\nabla_{x_{t}}\left\| 0-f(\hat{x}_{0}(x_{t})) \right\|_{2}^{2}$$

(33)

Here *f*($*$) is the governing equations.

Table S1 gives the algorithm of the physical-informed diffusion sampling algorithm.

## 3. **Training Protocols**

### **2.1. Comparison of training data formats**

The greater accuracy obtained for full-field flow data presented in the NPY format than that obtained using the GIF format can be evaluated based on the result of training prediction outputs for full-field *p* distribution image data presented in Figure S1, where the grayscale has been converted into two color schemes to more readily distinguish between positive and negative pressure regions. As observe, the GIF image presented in Figure S1(a) offers mottled predicted contours, while Figure S1(b) presents jagged pixel-based edges in the NPY image. These prediction result can be compared with the training data input associated with the original simulation result presented in Figure S1(c) and structurally interpolated result given in Figure S1(d). As observed, according to the interpolated results, NPY-based model owns a better prediction performance compared with the GIF-based trained model involving the flow data contours and airfoil envelopes.

### **2.2. Network training hyperparameters and experimental systems**

The hyperparameters employed when training the proposed PVDM framework are listed in Table S2. In addition, detailed information regarding the testing platforms employed for dataset generation, model training, and model testing are listed in Table S3.

### **2.3. Validation of the high spectral element method**

The high spectral element method was applied to increase the spatial resolution of grids via grid densification, where the *p*-th order was defined as *n* = 2, 3, 4, 5, 6. Accuracy of the process was evaluated by sampling the *C_L_* and *C_D_* coefficients extracted under some of the working cases, including NACA2230 and NACA4640, at different AoA values. The *C_L_* and *C_D_* error ratios obtained are plotted in Figure S2 as a function of the *p*-th order value.

As observed, the *C_L_* and *C_D_* error ratios generally decrease with increasing *p*-th order, and values less than 0.05 are generally obtained for *n* ≥ 4. This issue can be further evaluated based on the working case settings listed in Table S4, where the applied time step decreases substantially with increasing *p*-th order. However, the increasing accuracy also radically increases the force output file size and number of snapshot output documents. Accordingly, a *p*-th order of *n* = 4 is selected for all general flow domain calculation cases to balance computational accuracy with the time and storage costs.

### **2.4. Validation of *C_L_*/*C_D_* ratio token extraction process**

As shown in Figure S3 for *C_L_* obtained for airfoil NACA5431 over time at different AoA values, the aerodynamic load applied on the airfoil surface fluctuates over time. When reaching a steady-state force fluctuation, the lift and drag forces were extracted as the mean values, which can be plotted via time.

## 4. **Curve Fitting for Airfoil Inverse Design**

The applied Bezier curve fitting airfoil profile extraction process is illustrated in Figure S4. Here, the absolute values of *u*, *v*, and *p* components that are less than 0.01 are marked within the flow contours (Figure S4a). The maximum and minimum values of airfoil coordinates *X* are extracted for each row of airfoil body points, and the corresponding points are fitted by Bezier curves to obtain the airfoil profile (Figure S4b). The airfoil body is then predicted as the total area enclosed within the upper and lower boundaries of the fitted Bezier curves (Figure S4c). The predicted airfoil body is inserted into the simulated *u*, *v*, and *p* flow contours (Figure S4d).

## 5. **Full-field Flow Data Comparison**

The result presented in Figure 3c and d for the predicted and simulated pressure *p* distributions obtained for sample NACA2145 within 96 × 96 pixels global regions and 256 × 256 pixels airfoil-focused regions are expanded upon within Figs. S5 and S6, respectively, for all full-field flow components (*u*, *v* and *p*) over all 10 AoA values considered.

The result presented in Figure 4c and d for predicted and simulated velocity *u* distributions obtained for sample NACA5431 within 96 × 96 pixels global regions and 256 × 256 pixels airfoil-focused regions are expanded upon within Figure S7 and S8, respectively, for all full-field flow components (*u*, *v* and *p*) over all 10 AoA values considered.

The result shown in Figure 5c and d for predicted and simulated velocity *u* distributions obtained for sample NACA5431 within 96 × 96 pixels global regions and 256 × 256 pixels airfoil-focused regions are expanded upon within Figs. S9 and S10, respectively, for all full-field flow components (*u*, *v* and *p*) over all 10 AoA values considered.

Presented in Figure 6c and d, predicted and simulated velocity *u* distributions obtained for sample NACA5431 within 96 × 96 pixels global regions and 256 × 256 pixels airfoil-focused regions are expanded upon within Figure S11 and S12, respectively, for all full-field flow components (*u*, *v* and *p*) over all 10 AoA values considered.

The prediction errors obtained by the proposed approach must be evaluated carefully because the model does not directly predict binary pixels but rather *u*, *v*, and *p* distributions, which may be close to zero near to the airfoil wall boundary. Therefore, the present work applied peak signal-to-noise ratio (PSNR) and structural similarity index measure (SSIM) metrics for comparing the prediction results with the actual data. To this end, we first calculated the mean square error (MSE) as follows:

$$MSE=\frac{1}{mn}\sum_{i=0}^{m-1} \sum_{j=0}^{n-1} {[I\left( i,j \right)-k(i,j)]}^{2}$$

(35)

where the *i*-th and *j*-th pixel elements in the rows and columns of the distribution matrices extend to maximum values of *m* = *n* = 96. The MSE is then applied with the maximum pixel value in the matrix (*MAX_I_*) to obtain the PSNR value as follows:

$$PSNR=10\cdot\log_{10}\left( \frac{{MAX}_{I}^{2}}{MSE} \right)=20\cdot\log_{`10}(\frac{{MAX}_{I}}{\sqrt{MSE}})$$

(36)

The maximum possible PSNR value obtainable under the present circumstances is 49.771 *dB*. The SSIM is defined as a comparison of frames of pixel values *x* and *y* with the same dimensions from corresponding locations within two images as follows:

$$SSIM\left( x,y \right)=\frac{(2\mu_{x}\mu_{y}+c_{1})(2\sigma_{xy}+c_{2})}{(\mu_{x}^{2}+\mu_{y}^{2}+c_{1})(\sigma_{x}^{2}+\sigma_{y}^{2}+c_{2})}$$

(37)

Here, *μ_x_* and *μ_y_* are the mean pixel values of the respective frames, $\sigma_{x}^{2}$, $\sigma_{y}^{2}$, and $\sigma_{xy}$ are the variances of the pixel values, and the *c* values are constants applied to maintain stability, where $c_{1}={(k_{1}L)}^{2}$ and $c_{2}={(k_{2}L)}^{2}$, for *L* defined as the interval of pixel values, *k*_1_=0.01, and *k*_2_=0.02. Accordingly, the SSIM is a value in the range [0,1], where the structural similarity is perfect at an SSIM value of 1.

The date obtained for samples NACA2145 (sample 1), NACA5431 (sample 2), NACA7122 (sample 3), NACA5190 (sample 4) is listed in Table S5. To validate the robustness of the PVDM, Figure S13 and Figure S14 are presented to display the prediction performance, which are noted by the averaged MSE values.

# **Figures and Figure Captions**

#### **Table S1:** Physical-informed Diffusion Sampling Algorithm 1.

**Algorithm 1**

**Require:** Sampling step N, N-S Function *f*, Weights $\zeta_{ns}$ and Sampler $D_{\theta}\left( x,i \right)$

1: Sample $x_{N}\mathbb{\sim\mathbb{N}(}0,\sigma)$

2: **for** $i=N-1$ to 1 **do**

3: $\hat{d}_{i}\leftarrow D_{\theta}(x_{i},i)$

4: $\hat{x}_{1}\leftarrow\frac{1}{\sqrt{\bar{\alpha}_{i}}}(x_{i}+(1-\bar{\alpha}_{i})\hat{d}_{i})$

5: $\mathcal{z}\mathbb{\sim\mathbb{N}(}0,\sigma)$

6: $x_{i-1}\leftarrow\frac{\sqrt{\bar{\alpha}_{i}}(1-\bar{\alpha}_{i-1})}{1-\bar{\alpha}_{i}}x_{i}+\frac{\sqrt{\bar{\alpha}_{i}}\beta_{i}}{1-\bar{\alpha}_{i}}\hat{x}_{1}+\sigma_{i}\mathcal{z}$

7: $\mathbb{L}_{ns}=\left\| 0-f(\hat{x}_{1}) \right\|^{2}$

8: $x_{i-1}\leftarrow x_{i-1}-\zeta_{ns}\nabla_{x}\mathbb{L}_{ns}$

9: **end for** $x_{t-1}=\frac{\sqrt{\bar{\alpha}_{t}}\left( 1-\bar{\alpha}_{t-1} \right)}{1-\bar{\alpha}_{t}}x_{t}+\frac{\sqrt{\bar{\alpha}_{t}}\beta_{t}}{1-\bar{\alpha}_{t}}\hat{x}_{1}+\sigma_{t}\mathcal{z}$

10: **Return** $x_{1}$

#### **Table S2:** Network training hyperparameters.

| **Hyperparameters** | **Value** |
| --- | --- |
| Training data size  Batch size  Learning rate  Optimization algorithm  Iterations  Exponential Moving Average (EMA) decay  EMA start (iteration)  EMA update period (iteration)  DDPM timesteps  PDE Guidance start timesteps  NS constraint coefficient (for PDE guidance)  Condition dropout (for classifier-free guidance) | 17800  4  1e-4  Adam  100000  0.995  2000  10  256  100  1.0  0.1 |

#### **Table S3:** System information regarding experimental platforms.

| **Task** | **Software** | **Hardware** | **Runtime** |
| --- | --- | --- | --- |
| Dataset generation  Model Training  Inference | Nektar++/5.1.0  PyTorch in Python  PyTorch in Python | CPU (16 cores)  Nvidia A800 GPU  Nvidia A800 GPU | 9500 Hours  14 Hours  1 minute |

#### **Table S4.** Hyperparameters of working case settings for validation at the *p*-th order defined as *n* = 2,3,4,5,6 and corresponding output file size.

| **p’th order** | **Time step** | **Number of steps** | **Time length** | **Info_output step** | **Force output file size (*kb*)** |
| --- | --- | --- | --- | --- | --- |
| *n*=2 | 0.001 | 60000 | 60 | 2500 | 845 |
| *n*=3 | 0.0008 | 75000 | 60 | 3125 | 1056 |
| *n*=4 | 0.0005 | 120000 | 60 | 5000 | 1688 |
| *n*=5 | 0.0002 | 300000 | 60 | 12500 | 4220 |
| *n*=6 | 0.0001 | 600000 | 60 | 25000 | 8438 |

#### **Table S5:** MSE, PSNR and SSIM defining the prediction performance obtained for the three representative NACA airfoil samples discussed in Figure 3, 4, 5 and 6 (shown in the main text) over all of 10 AoA values are considered.

|  | **MSE** | **PSNR (*dB*)** | **SSIM** |
| --- | --- | --- | --- |
| Sample 1: NACA2145 | | | |
| u | 1.7×10^-5^ | 47.996 | 0.9966 |
| v | 1.1×10^-5^ | 49.771 | 0.9959 |
| p | 1.4×10^-5^ | 48.941 | 0.9946 |
| Sample 2: NACA5431 | | | |
| u | 1.078×10^-3^ | 29.861 | 0.7895 |
| v | 2.96×10^-4^ | 35.327 | 0.8436 |
| p | 3.87×10^-4^ | 34.202 | 0.8308 |
| Sample 3: NACA7122 | | | |
| u | 2.76×10^-4^ | 35.716 | 0.9064 |
| v | 7.3×10^-5^ | 41.439 | 0.9647 |
| p | 1.41×10^-4^ | 40.668 | 0.9586 |
| Sample 4: NACA5190 | | | |
| u | 3.4×10^-5^ | 45.317 | 0.99040 |
| v | 2.5×10^-5^ | 46.341 | 0.99420 |
| p | 1.08×10^-4^ | 42.410 | 0.99310 |
| Sample 5: NACA2827 | | | |
| u | 0.000012 | 49.5809 | 0.9969 |
| v | 0.000004 | 54.4822 | 0.9973 |
| p | 0.000008 | 51.9669 | 0.9968 |
| Sample 6: NACA3159 | | | |
| u | 0.000013 | 49.0816 | 0.9982 |
| v | 0.000008 | 51.1613 | 0.9968 |
| p | 0.000012 | 49.4814 | 0.9959 |
| Sample 7: NACA4711 | | | |
| u | 0.000027 | 47.7294 | 0.9931 |
| v | 0.000028 | 46.2378 | 0.9940 |
| p | 0.000032 | 49.1289 | 0.9920 |
| Sample 8: NACA7235 | | | |
| u | 0.000029 | 45.5776 | 0.9929 |
| v | 0.000011 | 50.0443 | 0.9935 |
| p | 0.000028 | 47.1119 | 0.9891 |
| Sample 9: NACA7340 | | | |
| u | 0.000034 | 44.9566 | 0.9937 |
| v | 0.000013 | 49.2220 | 0.9930 |
| p | 0.000040 | 45.4110 | 0.9857 |
| Sample 10: NACA7462 | | | |
| u | 0.000068 | 42.2265 | 0.9896 |
| v | 0.000034 | 45.2948 | 0.9860 |
| p | 0.000065 | 42.1562 | 0.9775 |
| Sample 11: NACA7551 | | | |
| u | 0.000028 | 45.8161 | 0.9948 |
| v | 0.000013 | 49.2550 | 0.9933 |
| p | 0.000035 | 45.5929 | 0.9868 |

#### **Figure S1.** Result of training prediction outputs for full-field p distribution data: a. original GIF-based training prediction output; b. NPY-based training prediction output; c. Structurally interpolated results; d. Primitive simulation results.

|  |  |  |  |
| --- | --- | --- | --- |
| **a.** GIF-based training prediction output | **b.** NPY-based training prediction output | **c.** Structurally interpolated results | **d.** Primitive simulation results |

#### **Figure S2.** *C_L_* and *C_D_* error ratios plotted versus the *p*-th order value of the high spectral element method for different airfoils: a. *C_L_* error ratio for NACA2230; b. *C_D_* error ratio for NACA2230; c. *C_L_* error ratio for NACA4640; d. *C_D_* error ratio for NACA4640.

#### **Figure S3.** Sampled lift force coefficient *C_L_* of airfoil NACA5431 versus solution temporal scale: *t*.

#### **Figure S4.** Bezier curve fitting process applied for extracting airfoil profile coordinates: a. airfoil coordinates meeting $0<\left| u \right|\cap\left| v \right|\cap\left| p \right|<0.01$ constraints; b. Bezier curve fitting of airfoil profile based on the maximum and minimum values of coordinates *X* observed for each row of airfoil body points; c. predicted airfoil body based on the upper and lower fitted boundaries; d. predicted airfoil body inserted within flow contours.

|  |  |
| --- | --- |
| **a.** Filtered airfoil coordinates | **b.** Bezier curve fitting |
|  |  |
| **c.** Fitted airfoil body | **d.** Inserted airfoil body in flow contours |

#### **Figure S5**. Predicted and simulated full-field flow components for sample NACA2145 within 96×96 pixels global regions.

**a.** *p* components

**b.** *u* components

**c.** *v* components

#### **111Figure S6.** Predicted and simulated full-field flow components for sample NACA2145 within 256 × 256 pixels airfoil-focused regions.

**a.** *p* components

**b.** *u* components

**c.** *v* components

#### **Figure S7.** Predicted and simulated full-field flow components for sample NACA5431 within 96 × 96 pixels global regions.

**a.** *p* components

**b.** *u* components

**c.** *v* components

#### **Figure S8.** Predicted and simulated full-field flow components for sample NACA5431 within 256 × 256 pixels airfoil-focused regions.

**a.** *p* components

**b.** *u* components

**c.** *v* components

#### **Figure S9.** Predicted and simulated full-field flow components for sample NACA7122 within 96 × 96 pixels global regions.

**a.** *p* components

**b.** *u* components

**c.** *v* components

#### **Figure S10.** Predicted and simulated full-field flow components for sample NACA7122 within 256 × 256 pixels airfoil-focused regions.

**a.** *p* components

**b.** *u* components

**c.** *v* components

#### **Figure S11.** Predicted and simulated full-field flow components *p* explained by streamlines for sample NACA5190 within 256 × 256 pixels airfoil-focused regions.

**a.** *p* components with streamlines

**b.** *u* components

**c.** *v* components

#### **Figure S12.** Predicted and simulated full-field flow components for sample NACA5190 within 256 × 256 pixels airfoil-focused regions.

**a.** *p* components

**b.** *u* components

**c.** *v* components

#### **Figure S13.** Predicted and simulated full-field flow components for randomly selected 4 samples (from sample 1 to sample 4) within 256 × 256 pixels airfoil-focused regions.

#### **Figure S14.** Predicted and simulated full-field flow components for randomly selected 4 samples (from sample 5 to sample 8) within 256 × 256 pixels airfoil-focused regions.
